# High-resolution analytical imaging and electron holography of magnetite particles in amyloid cores of Alzheimer’s disease

**DOI:** 10.1038/srep24873

**Published:** 2016-04-28

**Authors:** Germán Plascencia-Villa, Arturo Ponce, Joanna F. Collingwood, M. Josefina Arellano-Jiménez, Xiongwei Zhu, Jack T. Rogers, Israel Betancourt, Miguel José-Yacamán, George Perry

**Affiliations:** 1 Department of Physics and Astronomy, The University of Texas at San Antonio (UTSA), San Antonio, TX, 78249, USA; 2 School of Engineering, University of Warwick, Coventry, CV4 7AL, UK; 3 Department of Pathology, Case Western Reserve University, Cleveland, OH, 44106, USA; 4 Department of Psychiatry, Harvard Medical School, Boston, MA 02115, USA; 5 Department of Biology, The University of Texas at San Antonio (UTSA), San Antonio, TX, 78249, USA

## Abstract

Abnormal accumulation of brain metals is a key feature of Alzheimer’s disease (AD). Formation of amyloid-β plaque cores (APC) is related to interactions with biometals, especially Fe, Cu and Zn, but their particular structural associations and roles remain unclear. Using an integrative set of advanced transmission electron microscopy (TEM) techniques, including spherical aberration-corrected scanning transmission electron microscopy (Cs-STEM), nano-beam electron diffraction, electron holography and analytical spectroscopy techniques (EDX and EELS), we demonstrate that Fe in APC is present as iron oxide (Fe_3_O_4_) magnetite nanoparticles. Here we show that Fe was accumulated primarily as nanostructured particles within APC, whereas Cu and Zn were distributed through the amyloid fibers. Remarkably, these highly organized crystalline magnetite nanostructures directly bound into fibrillar Aβ showed characteristic superparamagnetic responses with saturated magnetization with circular contours, as observed for the first time by off-axis electron holography of nanometer scale particles.

Alzheimer’s disease (AD) is the most common cause of irreversible dementia among older people, affecting more than 5 million patients older than 65 years in the USA alone^1^. The exact mechanisms that trigger AD are not completely understood. Evidence suggests that AD is caused by a complex synergism between genetic predisposition, ageing, environmental, occupational, metal over-exposure and ancillary factors over a long period of time. Particularly, in advanced stages of AD it is evident there is a severe loss of neuronal connections as well as a high degree of formation and spread of abnormal filaments of amyloid-β (Aβ, forming amyloid plaques cores, APC), and tau protein (neurofibrillary tangles NFT) and altered levels of biometals and neurotransmitters^1^,^2^. Specific factors that promote accumulation and aggregation of amyloid-β into APC remain unclear. Several studies found connections with metal-catalyzed oxidative stress, high production of free radicals, reactive oxygen species and mitochondrial dysfunction^3^,^4^; genetic predisposition^5^, epigenetic factors^6^,^7^, and also activation of inflammatory pathways^8^, and other processes related to ageing.

Aβ is formed through the amyloidogenic pathway (sequential cleavage by β- and γ-secretase) of AβPP (amyloid-β protein precursor). The expression, processing and aggregation of Aβ are partially regulated by neurocortical metal ions (Zn, Fe and Cu) by modulating the translational and transcriptional control of AβPP, enzymatic activities of cell surface metalloproteinase family of desintegrin and metalloprotease (ADAM, which includes secretases), homodimerization of Aβ, and modulating the expression and activity of BACE1 and SOD1 through oxidative stress signaling^2^,^9^,^10^. Aβ_1−42_ possesses binding sites for Cu^2+^ and Zn^2+^ at physiological pH, but under acidic conditions Fe ions bind to Aβ, whereas Cu displaces Zn^9^. The dyshomeostasis in Cu, Zn and Fe levels and molecular interactions with Aβ may trigger its aggregation into fibrillar forms in APC^2^,^9^,^11^,^12^. Metabolism and transport of Fe is highly regulated at the translational level, controlling expression of corresponding Aβ, ferritin, transferrin receptor and ferroportin genes; nevertheless ageing and AD promote over-expression of ferritin and under-expression of transferrin, that in consequence may increase levels of Fe associated with neurofibrillary tangles and APC^9^,^10^,^13^. Interaction of metal ions with Aβ has been related to production or reduction of reactive oxygen species (ROS), causing a misbalance in neuronal redox potential and induction of oxidative stress with a significant role in progression of AD, whereas AβPP may have ferroxidase activity and interaction with ferroportin^14^. Consequently, alterations of homeostasis, transport and regulatory proteins of Fe, Cu and Zn ions are closely related with the progression and pathogenesis of AD, yet with all these changes the locations of metals in end-stage AD have not been clearly established.

Analyses and characterization of hybrid samples, in this case metallo-proteins, requires use of new advanced imaging techniques. By their size range, the interactions between proteins and metals occur at the nano-bio interface, comprising the dynamic physicochemical interactions between inorganic materials and biological components^15^. In particular, ultra-high resolution field-emission scanning electron microscopy (UHR FE-SEM) and spherical aberration-corrected scanning transmission electron microscopy (Cs-STEM) allow higher resolution and sensitivity than conventional imaging techniques^16^,^17^. Specifically, by using cold field-emission electron guns and improved electron column design STEM allows efficient collection of multiple elastic and inelastic signals (HAADF, EDX, EELS, holography) to obtain an advanced analytical imaging and quantitative characterization with atomic resolution of sensitive biological samples.

In this work, we present the analysis of amyloid plaque cores isolated from Alzheimer disease patients through an integrative set of advanced analytical electron microscopy techniques. Particularly, to determine the presence and chemical identity of inorganic materials associated with APC, confirming the presence of nanostructured aggregates of iron oxide, whereas other metals such as Cu and Zn were associated with Aβ fibers. Atomic-resolution aberration-corrected STEM imaging, coupled with energy dispersive X-ray spectroscopy (EDX) and electron energy loss spectroscopy (EELS) detectors allowed a precise identification and quantification of metals, confirming that iron-rich aggregates effectively had properties consistent with magnetite (Fe_3_O_4_). Magnetite nanoparticles located with APC showed a size range of 8–50 nm diameter with characteristic superparamagnetic responses as observed by off-axis electron holography. Particularly, holography of Fe_3_O_4_ particles revealed the phase contribution of the ferrite mean inner potential associated with the composition and density as circular contours. In conclusion, advanced electron analytical microscopy confirmed that the predominant form of iron within APC as nanostructured particles, and revealed their ultra-structural location, chemical identity and functional properties with high spatial sub-nanometer resolution, providing evidence of the metallo-biology implicated in iron accumulation and Aβ aggregation in Alzheimer’s disease and its connection to a dysregulated processing/transport of metal ions in human neurons.

## Results

### Isolation and microscopic detection of amyloid plaque cores

**Figure 1:**
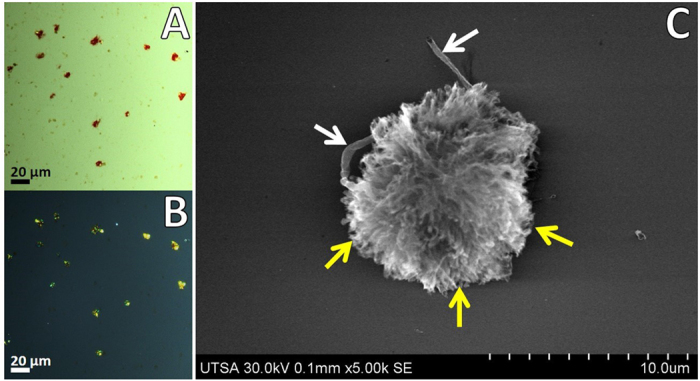
Imaging of APC through polarized light microscopy and UHR FE-SEM. APC were stained with Congo red and analyzed under polarized light for characteristic green birefringence of amyloid protein aggregates. (**A**) Transmitted light. (**B**) Polarized light. (**C**) High resolution imaging of APC by FE-SEM revealed the details of compact aggregates with fibrillar radial projections (yellow arrows) and presence of blood vessels (white arrows).

A definitive diagnosis of Alzheimer’s disease is made by neuropathological evaluation of postmortem autopsy of the most affected areas: the frontal and temporal lobes, and hippocampus^18^. We employed frozen brain tissues collected from patients that showed evidence for the presence of APC, demonstrated by pathological analysis with immunocytochemistry (Braak stage VI). Amyloid plaque cores were isolated from homogenized gray matter tissue by gradient ultracentrifugation^19^,^20^. The histopathological dyes Congo red and thioflavin S provided qualitative evidence for presence of APC in the gradient fractions. Figure 1A,B shows APC with Congo red staining under transmitted and polarized light, respectively. Additionally, enriched fractions of APC were sorted by size through FACS. Small fragments, cell debris and oversized aggregates were mostly removed, recovering principally APC of 5–30 μm by using a 100 μm nozzle for further analysis (Supplementary Fig. S1). This extensive isolation process ensured that only complete APC were employed during the high resolution analytical imaging.

### Imaging of APC by UHR FE-SEM and STEM

**Figure 2:**
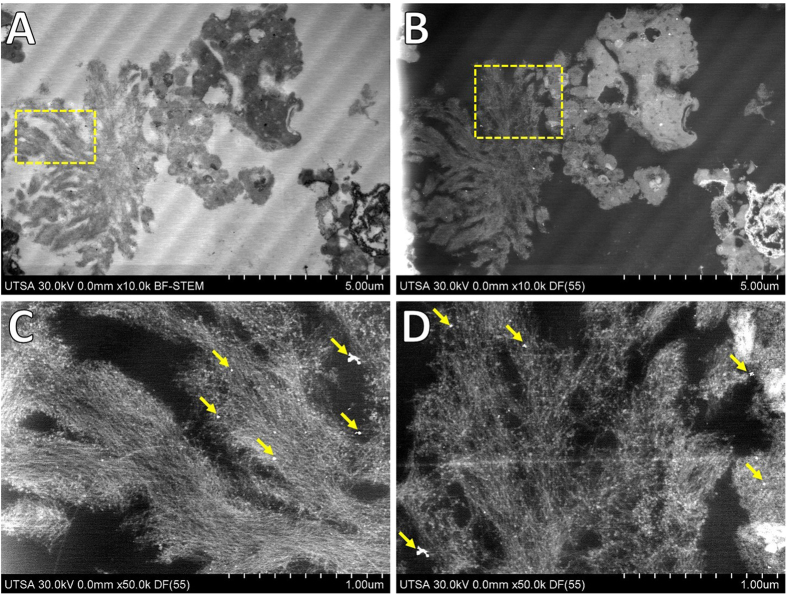
Imaging of ultra-thin sections of amyloid cores from AD. (**A**) Low voltage BF-STEM revealed the ultra-structure and arrangements of amyloid fibers. (**B**) Low voltage ADF-STEM showed presence of high-contrast areas and spots within amyloid cores. (**C**,**D**) High magnification ADF-STEM imaging revealing presence of electrodense aggregates linked to amyloid fibers, indicated with yellow arrows.

Besides histological identification of APC with polarized light, the amyloid cores are characterized by their fibrillar morphology. Aβ plaques have variable diameters from 1 μm up to 50 μm which are fibrillar or dense-cored^21^,^22^,^23^. Imaging of isolated APC through UHR FE-SEM revealed a compact dense plaque with fibrillar morphology (Fig. 1C). APC are described as pompoms showing protruding spikes or tufts, details of the fibrillar structures formed by Aβ from different sections of the APC are indicated with yellow arrows in Fig. 1C. APC were of 8–15 μm in diameter showing pompom-like morphology with elongated compact arrangement of filaments of 7–12 nm in diameter, with presence of some fractions of blood capillaries along the dense clump (white arrows in Fig. 1C). The presence of APC and tangles in AD cause a progressive neurodegenerative disease resulting in synaptic failure and neuronal death^24^,^25^,^26^,^27^. Also, a compromised integrity of intracellular vesicles is an early sign of Aβ pathogenesis which eventually may lead to neuronal cell death^28^. Altered levels of biometals have been related with neurogenerative diseases, especially in AD with accumulation of highly active iron ions (Fe^2+^). Particularly, this excess iron by interaction with Aβ cause its precipitation and also may lead formation of inorganic aggregates of recognized iron oxides observed in the human brain with variable arrangements: ferrihydrite (5Fe_2_O_3_ • 9H_2_O), haematite (Fe_2_O_3_), magnetite (Fe_3_O_4_), maghemite (γ-Fe_2_O_3_), wüstite (FeO) and goethite (α-FeO(OH))^29^. Ultra-thin sections of isolated APC showed presence of electrodense aggregates coupled to compact fibrillar arrangements (Fig. 2). The morphological phenotype of APC most present in advanced AD are dense-cored, with a compact central mass encircled by an outer compact layer of Aβ protruding as spikes^22^. Similar iron-based particles were previously observed in frozen, pelleted or homogenized human brain tissue^30^,^31^, but their association with cellular components remained unclear. Due to their nanometer-scale size, electron beam imaging of these iron-based materials is not possible in complete plaques, but using the ADF detector in STEM allowed us to locate these aggregates that presented higher contrast (due to *Z*^2^-contrast, *Z* is the atomic number) in comparison with the proteinaceous matrix formed by Aβ. Negative-staining enhanced the contrast and clearly revealed that the aggregates were linked to the Aβ fibers, identified by their characteristic fibrillary crossed structure of 7–12 nm in diameter.

### Aberration-corrected HAADF-STEM imaging

**Figure 3:**
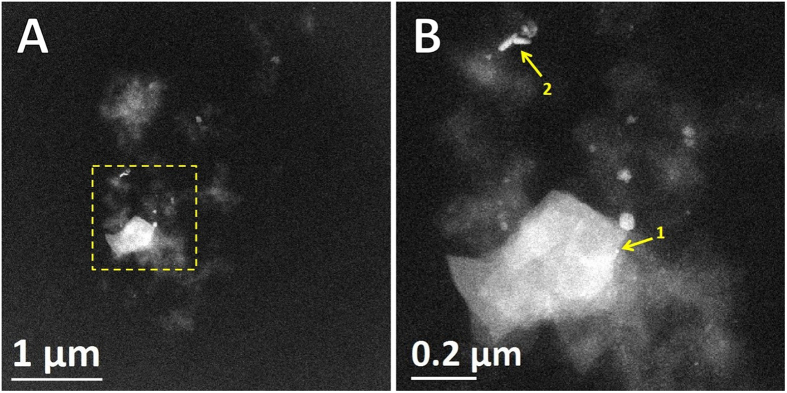
Aberration corrected-BF/HAADF-STEM imaging. APC were cut in ultra-thin sections of 90–95 nm for ultra-structural examination and location of electrodense materials, samples were not stained with heavy metals to avoid noise signals. (**A**) Low magnification of APC thin sections identifying presence of electrodense areas and spots. (**B**) High magnification STEM of selected area in (**A**), locating different high-contrast spots corresponding to particles into APC (yellow arrows, labeled 1 and 2). Imaging was carried out with JEOL ARM-200F operated in STEM mode at accelerating voltage of 80 kV, to avoid or reduce radiation damage of protein-based aggregates.

Use of advanced analytical electron microscopy techniques, particularly spherical aberration corrected scanning transmission electron microscopy (Cs-STEM) coupled with a high-angle annular dark-field (HAADF) detector, were used to gain a deep understanding of the ultrastructure and chemical nature of the metals in APC. HAADF-STEM images are directly interpretable by their *Z*^2^-contrast, heavier atoms present higher electron scattering and contrast than lighter atoms^16^,^17^. HAADF-STEM images in Fig. 3 clearly revealed the presence of high-contrast areas corresponding to APC. Ultra-thin sections of APC were not post-fixed (OsO_4_) nor negatively stained with the heavy-metals commonly employed in electron microscopy of biological samples, in order to analyze the metals contained *in situ* and avoid any background or noise signals which could obscure their characteristic signals. The contrast in HAADF mode originated only from scattering of elements present in APC. Among the cores, some round and elongated structures provided an extraordinary contrast (arrows in Fig. 3B), similar electron dense regions in APC have previously been observed and identified to contain Fe, Al, Si, Cu, and trace amounts of other inorganic elements^26^,^32^,^33^. Previously we showed the affinity and metal binding activity of APC by Raman spectroscopy, showing Zn^2+^ and Cu^2+^ coordinated to His side chains of Aβ^34^. Additional evidence of the high affinity of Aβ for metals (Cu^2+^) arises from *in vitro* fluorescence and electron paramagnetic resonance spectroscopies^35^. Aggregation of Aβ increases in a significant way when metal ions are present, causing precipitation and ultimately formation of synthetic Aβ fibers highly enriched in metals^36^,^37^.

### Atomic resolution STEM of APC-metal particles

**Figure 4:**
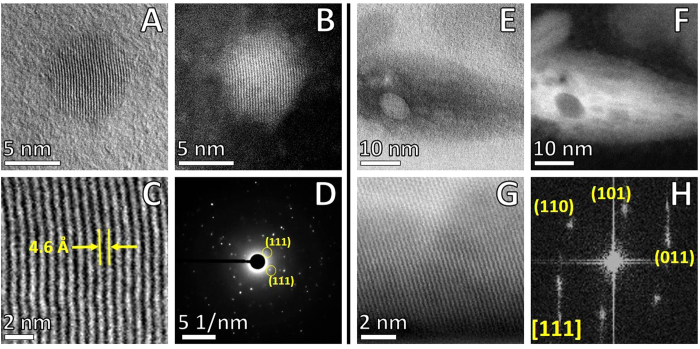
Atomic resolution Cs-BF/HAADF-STEM imaging. High-contrast spots corresponding to iron oxide particles within APC (yellow arrows, labeled as 1 and 2 in Fig. 2). (**A**) BF-STEM spherical Fe nanoparticle. (**B**) HAADF-STEM spherical Fe nanoparticle. (**C**) Detail of surface of Fe nanoparticle. (**D**) Nano-beam electron diffraction. Elongated particles: (**E**) BF-STEM non-spherical Fe nanoparticle. (**F**) HAADF-STEM non-spherical Fe nanoparticle. (**G**) Detail of periodic surface of Fe nanoparticle. (**H**) Fast Fourier Transform (FFT) pattern of selected area in (**G**). Imaging with JEOL ARM-200F, operated in STEM mode at accelerating voltage of 80 kV.

HAADF-STEM revealed details of size, shape and arrangements of inorganic materials into APC. Figure 4A,B shows high magnification BF/HAADF-STEM of a spherical particle with a diameter of 8 nm, where it is possible to distinguish atomic columns with high arrangement into a crystalline nanostructure. Detail of HAADF-STEM at atomic resolution in Fig. 4C revealed an interplanar spacing of 0.46 nm, which is close to the cubic crystal structure of magnetite (Fe_3_O_4_). Nano-beam diffraction and FFT pattern of the selected area showed a bcc (body-centered cubic) system oriented in the (011) beam direction. Besides individually spherical particles, we identified the presence of elongated nanostructures, which showed similar bcc crystalline structures and interplanar spacing. High magnification STEM revealed that these metal-rich aggregates could be formed by several small particles (Fig. 4E,F). These irregular morphologies can be derived from an incomplete crystallization and agglomeration effects of iron oxide particles. Magnetite (Fe_3_O_4_) is the most stable iron oxide formed by hydrolysis of Fe^2+^ and Fe^3+^ ions, in the presence of OH^−^ precipitating by partial reduction from ferric to ferrous ions at room temperature^38^,^39^, even though the mineralization mechanism of magnetite formation in human brain is still unknown. Synthetic magnetite-Aβ caused severe deterioration of *in vitro* clustered neuronal circuits from rat, by observing alterations in functional connectivity and damage^39^. Fe ions and high levels of OH^•^ radicals have been associated with increased oxidative stress by overproduction of reactive oxygen species (ROS: O_2_^−^, OH^•^ and H_2_O_2_) which may trigger neuronal damage in AD^40^. The development of AD occurs over a long period, our findings suggest Fe does not bind to Aβ in plaques, it was accumulated preferentially into aggregates.

### EELS and EDX of APC

**Figure 5:**
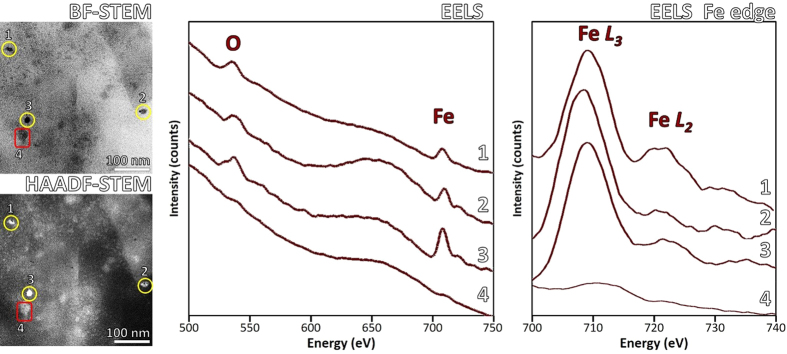
Identification of particles analyzed by EELS microanalysis. Electron energy loss spectroscopy of high-contrast individual particles located in Cs-BF/HAADF-STEM imaging. Corresponding EELS spectra of points indicated as (1), (2) and (3) and area in (4) indicating presence of characteristic oxygen and iron peaks. Normalization of background signal allowed identification of Fe *L*_3_ and Fe *L*_2_ edge peaks.

**Figure 6:**
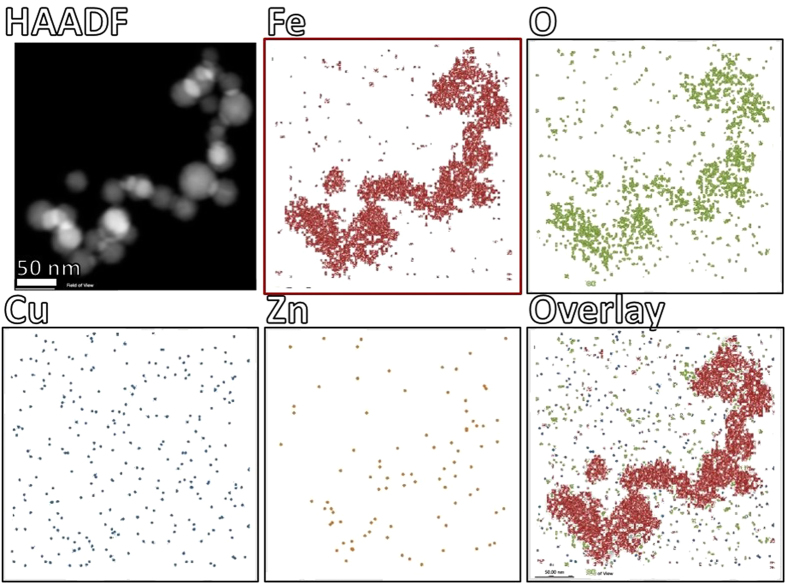
Identification of particles analyzed by EDX microanalysis. EDX mapping of Iron (Fe, red), Oxygen (O, green), Copper (Cu, blue) and Zinc (Zn, yellow) over the area identified by HAADF-STEM, and merge of Fe and Cu. EDX spectra indicate location of characteristic X-ray peaks of the elements present in the ultra-thin sections. Acquisition with Octane Silicon Drift Detector (SDD) and TEAM EDS Analysis System (EDAX).

Cs-STEM in combination with electron energy loss spectroscopy (EELS) and energy dispersive X-ray spectroscopy (EDX) provided chemical and electronic state information of elements present within APC. Figure 5 shows BF/HAADF-STEM and the corresponding EELS spectra of four different regions of the APC. Areas 1, 2 and 3 were selected due to their higher contrast in the area under analysis, whereas STEM revealed the crystallinity and arrangement. Corresponding EELS spectra clearly revealed presence of peaks located at 530 and 708–710 eV, analysis and integration with DeConvEELS (GATAN) identified that peaks corresponded to O*_K_* and Fe_*L*3_ with characteristic peaks centered at 532 and 708 eV, respectively. In contrast, EELS spectra of area 4 adjacent to the nanoparticles, did not present Fe nor O peaks even though it presented contrast in STEM imaging. Background substracted spectrum showed presence of Fe_*L*3_ and Fe_*L*2_ peaks in areas 1–3 but not in 4. Previously, EELS of complete APC confirmed the presence of Fe, but high scattering signals from protein matrix and carbon support prevented clear identification of Fe^2+^ and/or Fe^3+^ which would have permitted distinction between mixed valence magnetite and maghemite^26^. Complementary to EELS, the corresponding EDX spectra for the same areas under analysis were acquired. Corresponding characteristic peaks for O at 0.523 keV and Fe (0.705 and 6.403 keV) were identified in the spectra, confirming that elements under analysis effectively were iron oxide particles as observed by HAADF-STEM and nano-diffraction. Control area (#4) did not show sufficient counts for Fe, but clearly showed presence of small amounts of Cu 1.30%w (0.30%at) and 0.19%w (0.04%at) of Zn (Supplementary Fig. S2). Both EELS and EDX are coupled with a scanning focused electron probe obtaining analytical information for chemical identification with high spatial resolution. The size of the probe determined the quality of spectra obtained, under the conditions employed it was 0.8 Å. Figure S2 shows BF/HAADF-STEM of the areas analyzed where it was possible to see the points where the electron probe was focused during EELS and EDX acquisition after a long period of exposure, confirming that EELS and EDX spectra acquired corresponded to signals from the nanostructured particles. EDX acquired under scanning mode generated a map of the spatial distribution of the inorganic elements in the APC core. Spectral mapping of Fe and O clearly co-localized with high-contrast particles observed by HAADF-STEM (Fig. 6). In contrast, maps of Cu and Zn showed a more disperse distribution throughout the thin-section, indicating that the nanoparticulate material is adjacent (rather than coincident) with Cu and Zn and exclusively iron oxide in the Aβ compact matrix within the limits of detection. Integration of characteristic peaks showed that sample contained 24.25% of C, 67.54% of O, 4.82% of Fe, 2.98% of Cu and 0.42% of Zn, expressed as normalized weight percentage (Supplementary Fig. S3). At the molecular level the binding of oxidized and reduced ionic forms of Cu, Fe and Zn to Aβ occurs through side chains of Asp1, Ala2, Arg5, His6, Ser8, Tyr10, Glu11, His13 and His14^9^,^41^. Residues 1–17 of Aβ are disordered, while 18–42 domain form a β-strand-turn-β-strand motif which through intermolecular interactions assemble into protofilaments^42^. NMR spectroscopy and circular dichroism in combination with MD simulations previously revealed that the N-terminal of Aβ adopts different conformational steps depending on surrounding media, whereas the C-terminal turns from α-helix to beta conformation^43^. The dynamic structure and high mobility of the Aβ C-terminal explains the versatility to bind Cu, Fe and Zn ions under different physicochemical conditions (neutral or acidic), which ultimately may adopt a stable conformation upon binding with metallic precursors to assemble into protofibrillar structures forming APC. One of the most striking findings here is that the majority of the iron is not bound to Aβ fibers isolated from brain, consistent with our previous Raman scattering and electron tomography analysis of APC^26^,^34^.

### Electron Holography

**Figure 7:**
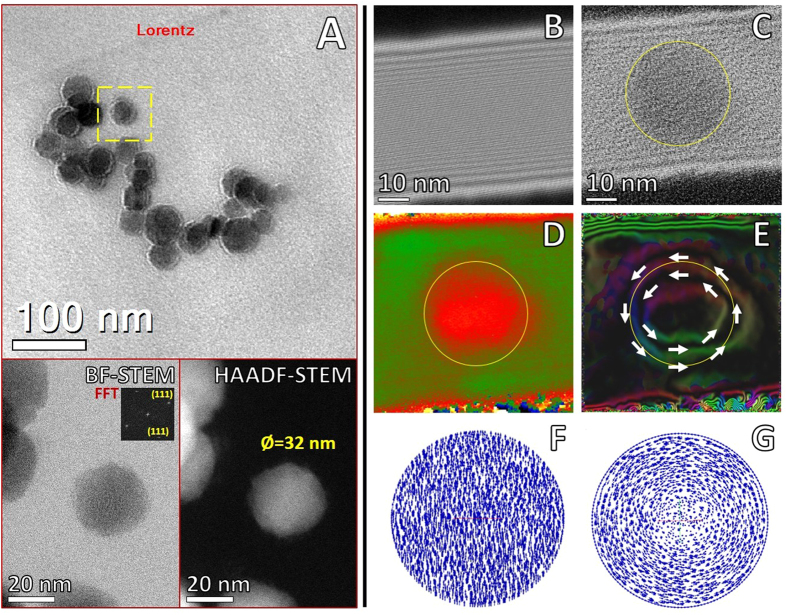
Off-axis electron holography of magnetic fields. (**A**) Lorentz microscopy of magnetite particles, complementary high magnification BF/HAADF-STEM of individual particles confirmed crystallinity and arrangement of 32 nm Fe_3_O_4_ nanoparticle. Off-axis electron holography revealed magnetic fields of magnetite particles. (**B**) Reference hologram, (**C**) Object hologram of particle indicated in **(A)**, the contour of nanoparticle was indicated with a yellow circle. (**D**) Magnetic contribution to the phase shift, (**E**) Colored magnetic phase contours around individual Fe_3_O_4_ particle, the arrows show the direction of magnetization. Micromagnetic calculations based on the dynamic magnetization by finite element method for spherical Fe_3_O_4_ nanoparticles for analysis of the direction of magnetization, (**F**) Saturated magnetization pointing along the magnetic anisotropy direction, (**G**) Saturated magnetization with circular orientation around middle zone.

One of the major current challenges in nano- and biosciences is the development of methods to measure magnetic fields at nanometer scale. Electron holography provides quantitative information about electron-transparent thickness (mean inner potential), electrostatic and magnetic contributions, as well as lattice distortion of samples in an intuitive form, allowing the visualization of structural, chemical and functional information of nanomaterials at the nanoscale with high spatial resolution^44^,^45^. Initially, Lorentz microscopy and medium resolution off-axis electron holography allowed location of magnetic Fe_3_O_4_ nanoparticles within APC (Figs 7A and S4). The objective dual lens was adjusted according to specific calibration settings for the JEM-ARM200F Atomic Resolution analytical Microscope, operated in TEM mode at 200 kV accelerating voltage^46^. Interference holograms of vacuum reference and specimen waves were acquired at 35 V of the biprism. Hologram phase, magnetic contours were obtained by subtracting interference fringes of the reference wave from specimen waves. Lorentz lens TEM conditions showed evidence of magnetic response in their remanent state (Fig. 7A). High-resolution electron holography of an individual Fe_3_O_4_ nanoparticle of 32 nm in diameter located within APC is presented in Fig. 7A lower part. The retrieved unwrapped hologram phase, magnetic contribution to the phase shift and corresponding magnetic contours were obtained by processing and reconstructing the interference fringes of reference waves with HoloWorks v.5.0 (Gatan Software DigitalMicrograph)^47^ (Fig. 7B,C). Individual magnetite particles showed superparamagnetic behavior characteristic of magnetite in this size range at 300 K. Our study demonstrated the first direct observation of magnetism of individual Fe_3_O_4_ nanoparticles directly bound within APC, in comparison with previous studies that employed SQUID magnetometry of intact or powdered and pelleted brain tissues from AD where the spatial relationship of the magnetite with APC could not be tested. In conjunction with STEM, we can confirm that the magnetic responses arise exclusively from magnetite embedded into APC, even though presence of iron biomineralization has been observed in other brain areas.

## Discussion

Amyloid proteins are identified by their high affinity to Congo red and the resulting characteristic green birefringence by polarizing microscopy, which is an anisotropic optical effect produced when bound to amyloid proteins^21^,^48^. These dyes bind with high specificity by interactions with positively charged residues (His, Arg and Lys) located on the vicinity groove of stacked β-sheets, without conformational alteration of 3D structure of protein fibers. Extracellular amyloid plaques and neurofibrillary tangles are both present in AD. The precise mechanisms of pathogenesis of APC is not clearly established, but in some steps it involves sorting to multivesicular bodies with intracellular growth of Aβ fibrils which over time infiltrate to multivesicular bodies promoting neuronal death, and are finally released to the extracellular space^28^,^49^. APC isolated from AD patients showed pompom-like structure of 5–15 μm with fibers protruding in all directions, their high stability notably resisted all purification steps and storage before and during analysis, some portions of vesicular bodies were also observed in APC.

Aberration corrected HAADF-STEM was employed for advanced analytical imaging of ultra-thin sections (95 nm thickness) of APC, since previous attempts with complete APC presented difficulties for full chemical and structural identification of the metals present. This incoherent imaging technique provides much higher resolution (sub-Angstrom) than conventional phase-contrast TEM^16^,^17^, and by coupling with *in situ* spectroscopic analysis allows an integrative imaging-quantification characterization platform of hybrid samples at high spatial resolution. Previous studies identified iron-rich materials in amyloid plaque cores identified as magnetite (Fe_3_O_4_) or maghemite (γ-phase, Fe_2_O_3_), but discrimination between these two ferrimagnetic Fe phases was not possible due to high similarities in crystalline structure^26^. Also bulk analysis showed iron oxide particles extracted from solubilized brain tissue having ferrimagnetic properties, where sufficient crystallographic information was obtained to positively identify both magnetite and its oxidation product maghemite^30^,^31^. Iron is transported by transferrin and stored by ferritin as a hydrated ferrihydrite-like iron oxide in a cavity approximately 8 nm in diameter. The mineral phase and crystalline quality may vary according to the subtype of ferritin and the health and age of an individual^50^. Remarkably, ferritin-bound iron accumulates in different areas of the brain in ageing, but the factors triggering this phenomenon remain unknown^51^. The hypothesis that Aβ is responsible for localized elevation of redox-active Fe^2+^, and thereby increased oxidative damage has received strong support from recent work confirming that iron in solution and ferric iron oxide nanoparticles are chemically reduced at physiological pH in the presence of aggregating Aβ_1−42_^37^,^52^. Also, AD patients have shown an increased expression of mitochondrial ferritin in response to proinflammatory cytokines^53^. Technical obstacles, particularly sample preparation/processing provided limited understanding of iron brain processing in APC^51^.

In this work, advanced analytical imaging of Fe nanoparticles directly associated to the cores of Aβ was accomplished at sub-nanometer resolution by use of spherical aberration-corrected STEM coupled with off-axis electron holography to directly reveal magnetic properties of magnetite particles, chemical composition and metal distribution in APC sections, with additional versatility from the use of low-voltage doses to reduce radiation damage to the proteins but keeping high spatial resolution of the magnetite nanostructures. By the size range of Fe_3_O_4_ particles (8–50 nm in diameter) and association with Aβ, this indicated that the iron oxide nanoparticles present in APC cannot be cleared by neurons or microglia cells, which may promote their accumulation. The origin of these magnetite particles might be related to ferritin with a 8 nm core^54^, that afterwards aggregate forming larger aggregates or could be formed by the redox properties of Aβ. Also, APC are tightly enveloped by microglia and astrocytes forming a barrier influencing plaque composition, axonal dystrophy and neurotoxicity^27^. The cytotoxic effects of iron oxide nanoparticles have been extensively studied. Upon exposure to iron oxide nanoparticles, cells may suffer membrane damage, impaired mitochondrial function, inflammation and apoptotic responses, chromosome condensation, DNA damage, and generation of ROS^55^. The Fe nanoparticles tested were coated with inorganic surfactants; bio-compatible compounds (proteins, peptides, carbohydrates or zwitterionic agents) can effectively reduce their toxicity. Like in the case of poly-lysine coated magnetite particles, they show no observable toxic effects after 24 h dose on primary Schwann cells of the peripheral nervous system and human neuroblastoma cells (SH-SY5Y)^56^. Some cytotoxic effects of magnetic nanoparticles can be attributed to chemical coating and by-products and not directly to the metal core, although long term effects in neurons and brain are unknown. Similarly, magnetite particles interacting with *in vitro* neuronal networks showed no side effects, but when combined with Aβ caused significant deterioration in neuronal functions and connectivity^39^.

In a previous study, we showed iron aggregates in complete APC were magnetite (Fe_3_O_4_) or maghemite (γ-phase, Fe_2_O_3_), but specific discrimination between the two Fe phases was not possible due to high similarities in their crystalline structure^26^. Also, EELS on complete APC precluded determining the chemical state of magnetite/maghemite nanoparticulate inclusions. Synchrotron X-ray microscopy was able to effectively identify the presence of iron foci in the form of magnetite and ferrihydrite-like phases in APC-rich superior temporal gyrus, but with a limitation of 5 μm spatial resolution and an embedding method that did not facilitate direct correlation with the APCs.

In this study, EELS and EDX performed in STEM mode on transversal ultrathin-sections allowed us to focus the coherent electron beam to a single point (0.8 Å), obtaining specific information about the elemental composition with characteristic peaks for Fe and O generated from the specific position selected with the electron beam, confirming that high-contrast Fe particles in APC are consistent with the magnetite phase. Selected area EELS analysis in the region 700–740 revealed the Fe 2*p L*_2,3_ edge with high resolution and sensitivity (Fig. 5). Spectra excluded Fe as a major component bound to Aβ, showing typical shape for a mixed valence (Fe^2+^/Fe^3+^) with Fe *L*_2_ around 708 eV and Fe *L*_3_ at 720–723 eV^57^, confirming identity of Aβ magnetite (Fe_3_O_4_) particles in comparison with α-Fe_2_O_3_ and γ-Fe_2_O_4_ that present an additional edge before *L*_3_. Adjustments to the configuration of the TEM to obtain electron holograms at specific voltages allowed fine-tuning of the field of view from μm to nm scale^46^. Magnetite particles showed superparamagnetic behavior, with vanishing influence of the magnetic contribution on the reconstructed phase, which allowed the mean inner potential to work as the prevailing signal contribution in the form of circular contours (Fig. 7E). This observation indicated a phase shifting proportional to thickness variations along the nanoparticle direction parallel to the incident electron beam. Additionally, since electromagnetic fields are present in the phase shift, the electrostatic and magnetic contributions must be separated; holograms with constant thickness do not produce fringes within nanoparticles whereas observations of fringes clearly reveal magnetic lines of force^45^. The reconstructed phase showed fringe contours (Fig. 7D,E), which are produced mainly by contributions from the mean inner potential with a vanishing contribution from the magnetic response of the Fe_3_O_4_ nanoparticles. The magnetic response of iron oxide is related to nanoparticle size; specifically the critical diameter for single domain particle formation (*D*_sd_) of Fe_3_O_4_ is ~80 nm at room temperature^58^. Whereas, particles of smaller size (25–30 nm) approach the lower critical diameter for superparamagnetic response (*D*_sp_) characterized by a fast flipping of the magnetization vectors presenting vanishing magnetization values^58^,^59^. Micromagnetic simulations of the equilibrium magnetization of Fe_3_O_4_ particles were computed to analyze the direct visualizations by holography. For particles with diameter <80 nm (Fig. 7F) the saturated magnetization pointed along the magnetic anisotropy direction, in contrast with particles >80 nm that showed a transition from single domain to multi-domain state characterized by magnetization along the magnetic anisotropy direction at the center and a circular orientation around the edges (Fig. 7G). Electron holography and micromagnetic simulations helped to reveal the superparamagnetic response of iron oxide particles present in the APC ultrathin sections from AD cases. Abnormalities in brain iron metabolism are linked to neurodegenerative diseases, the relation between iron overload and AD is subject of debate, but strongly supported by post-mortem evidence of elevated iron in brain regions^60^ and by evidence from MRI observations in AD patients^51^. These observations open the potential to develop novel non-conventional magnetic resonance imaging by using superparamagnetic properties of Fe_3_O_4_ particles as imaging markers for early diagnosis and monitoring in AD patients^61^,^62^,^63^.

## Conclusions

Metals play a significant role in neurobiology, particularly in neurodegenerative diseases. Iron, copper and zinc bind to Aβ, these interactions result in aggregation and precipitation into APC. High affinity of Aβ for metal ions is correlated with accumulation of Fe and their eventual aggregation into more stable particulate forms. These events are a possible mechanism of neurons associated to mitigate increased oxidative stress and misbalance of brain metals.

Using an integrative set of advanced analytical electron microscopy techniques allowed identification and quantification of the amounts of Fe, Cu and Zn present within APC. Cu and Zn were directly associated with Aβ fibers, while the majority of Fe was essentially independent from the fibers. Remarkably, iron oxide nanoparticles were highly structured and crystalline with 8–50 nm in diameter within the APC but separate from the fibers. Ultrastructural location, atomic arrangement and chemical composition of Fe aggregates as magnetite (Fe_3_O_4_) were confirmed by aberration-corrected STEM, electron nano-diffraction and *in situ* spectroscopy techniques (EELS and EDX). Interestingly, Fe_3_O_4_ present within APC showed a superparamagnetic response as observed by off-axis electron holography. The magnetic contours of magnetite corresponded to the mean inner potential and no lines of magnetic force were observed, which in fact is in agreement with the lower critical size for the superparamagnetic response and consistent with our previous observations from the collective content of APC. The presence of magnetic nanoparticles associated with AD reveals the importance of metallobiology of human neurons, as a key point for identification of misbalanced processing/transport of metal ions in neurodegenerative diseases.

## Methods

### Tissue source

Human brain tissue was obtained, with informed written consent of relatives from autopsy AD patients for neuropathology examination. All protocols used in this study were approved by the Bioethics Committee (Department of Pathology, Case Western Reserve University). All methods were carried out in accordance with the approved guidelines and regulations.

### Isolation of Amyloid Plaque Cores (APC)

All chemicals were of ACS or molecular biology grade and buffers prepared with fresh ultrapure water (MilliQ, Millipore). APC were isolated using a modified reported method^19^,^20^. Brains of patients diagnosed with Alzheimer (78 and 89 years old, Braak stage VI) were removed at autopsy (5 h postmortem), divided in half and cut into 1 cm slices (one hemisphere fixed with 10% para-formaldehyde and the other was stored at −70 °C). Sections of frozen tissue were thawed, and then grey matter from frontal and temporal lobes was cleaned by removing blood vessels, meninges and white matter. Grey matter was fined minced with a razor blade and dissolved for 2 h in homogenization buffer (2% SDS, 50 mM Tris, pH 7.6) adding a 5× v/w ratio of buffer. Tissue was homogenized in a Dounce glass homogenizer and then heated at 95 °C for 10 min. Homogenate was filtered through nylon mesh of 110 μm removing cell debris and immediately centrifuged at 1000 rpm for 30 min. Pellets were resuspended in wash buffer (1% SDS, 150 mM NaCl, 0.02% NaN_3_) and centrifuged again at 1000 rpm for 15 min. Supernatant was removed and pellets resuspended in small volume of wash buffer and passed through nylon mesh of 35 μm. A non-continuous gradient (1.2, 1.4, 1.6 and 1.8 M sucrose in buffer 1% SDS, 50 mM Tris, pH 7.6) was prepared in 32 ml thickwall polycarbonate tubes and adding 1–2 ml of concentrated sample. Tubes centrifuged at 72,000 g for 1 h at 10 °C using a Beckman L8–80 M ultracentrifuge and SW28 Ti rotor. All interfaces were collected, diluted 5× in wash buffer and centrifuged at 1500 rpm for 30 min. Finally pellets were resuspended in wash buffer and stored at 4 °C for further analysis. APC were sorted with BD FACS-Aria II by forward angle light-scattering and auto-fluorescence, using a 100 μm nozzle to recover 5–30 μm particles and removing small debris and large aggregates. Finally, APC were concentrated by centrifugation.

### Optical microscopy

Microscopy detection of isolated APC was performed by Congo red staining. 2–3 μl of each sample were placed on a positively charged microscope slide and dried at 100 °C for 10 min. Slides were stained for 30 min with Congo red solution (1% w/v in 80% ethanol, with 0.01 M NaOH), rinsed with 70% ethanol, 95% ethanol, xylenes and air dried. Coverslip was placed with help of water based mounting media. Slides were analyzed by polarization microscopy in a ZEISS AXIO A1 Imager to observe birefringence (green color) characteristic of APC stained with Congo red.

### Sample processing for electron microscopy

Samples enriched in APC were washed twice with sterile filtered PBS buffer and centrifuged at 3,000 rpm for 15 min. APC were dehydrated through a graded ethanol concentrations (50, 75, 95 and 100%) for 15 min each. 20 μl of cores in 100% ethanol were mounted on ultraflat silicon wafer in 5 × 5 mm chips (Ted Pella) and critical point dried for 16 cycles at slow speed (Leica EM CPD300). Ultha-thin sections of APC were obtained from a concentrated sample centrifuged at 10,000 rpm for 15 min and fixed with 4% formaldehyde/1% glutaraldehyde for 1 h at room temperature. Immediately, samples were rinsed two times with ddH_2_O for 15 min, continuing with dehydration for 15 min with 50, 75, 95 and 100% ethanol series solutions, respectively. APC were washed once in propylene oxide to remove ethanol, following with infiltration with 50% LX112 resin (Ladd Research) in propylene oxide for 1 h. Finally, samples were infiltrated with 100% LX112 resin and cured for 48 h at 65 °C. Ultrathin sections (95 nm) were cut with Leica Ultracut ultramicrotome using a 45° diamond knife (Diatome). Ultrathin sections were mounted on 300 mesh regular nickel grids (Electron Microscopy Sciences).

### Advanced electron microscopy: UHR FE-SEM and Cs-STEM

UHR FE-SEM and low voltage BF/DF-STEM imaging were carried out with a S-5500 In-Lens UHR FE-SEM (HITACHI High Technologies) coupled with Duo BF/DF-STEM detector and a solid state EDX detector (Bruker) operated with an accelerating voltage of 30 kV. Micrographs were recorded and analyzed with Quartz PCI. Spherical aberration-corrected scanning transmission electron microscopy (Cs-STEM) with JEM-ARM200F operated in STEM mode at accelerating voltage of 80 or 200 kV, coupled with energy dispersive X-ray spectrometer (EDAX) and GIF Quantum Energy Filter (GATAN). Micrographs were recorded and analyzed with Gatan Microscopy Suite (DigitalMicrogragh). X-ray microanalysis processed with TEAM EDS Analysis System (EDAX) and EELS processed with DeConvEELS and EELSTools (DigitalMicrogragh plugins).

### Off-axis electron holography

Electron holography was performed with JEM-ARM200F operated in TEM mode at accelerating voltage of 200 kV. Holograms were acquired at a voltage of biprism of 35 V, objective lens of 7.98 V with field of view of 65 × 65 nm at 300 K. Processing and reconstructing of the interference fringes and reference waves with HoloWorks v.5.0 (Gatan Microscopy Suite, DigitalMicrograph) using a fringe contrast of 26%, fringes width (W) of 52 nm and fringes spacing (σ) of 0.36 nm.

## Additional Information

**How to cite this article**: Plascencia-Villa, G. *et al*. High-resolution analytical imaging and electron holography of magnetite particles in amyloid cores of Alzheimer’s disease. *Sci. Rep*. **6**, 24873; doi: 10.1038/srep24873 (2016).

## Supplementary Material

Supplementary Information
