# Supplementary material for: High-resolution analytical imaging and electron holography of magnetite particles in amyloid cores of Alzheimer’s disease
Source: Sci Rep. 2016 Apr 28;6:24873. doi: 10.1038/srep24873 (PMC4848473; doi:10.1038/srep24873)
Supplement: Supplementary Information [file srep24873-s1.pdf]

High-resolution analytical imaging and electron holography of magnetite particles in amyloid cores of Alzheimer´s disease

Germán Plascencia-Villa[1], Arturo Ponce[1], Joanna F. Collingwood[2], M. Josefina Arellano-Jiménez[1], Xiongwei Zhu[3], Jack T. Rogers[4], Israel Betancourt[1], Miguel José-Yacamán[1] and George Perry[5]

[1] Department of Physics and Astronomy, The University of Texas at San Antonio (UTSA), San Antonio, TX, 78249, USA.
[2] School of Engineering, University of Warwick, Coventry, CV4 7AL, UK.
[3] Department of Pathology, Case Western Reserve University, Cleveland, OH, 44106, USA.
[4] Department of Psychiatry, Harvard Medical School, Boston, MA 02115, USA.
[5] Department of Biology, The University of Texas at San Antonio (UTSA), San Antonio, TX, 78249, USA.
Correspondence and request for materials should be addressed to GPV. (email: german.plascenciavilla@utsa.edu)

Supplementary Information

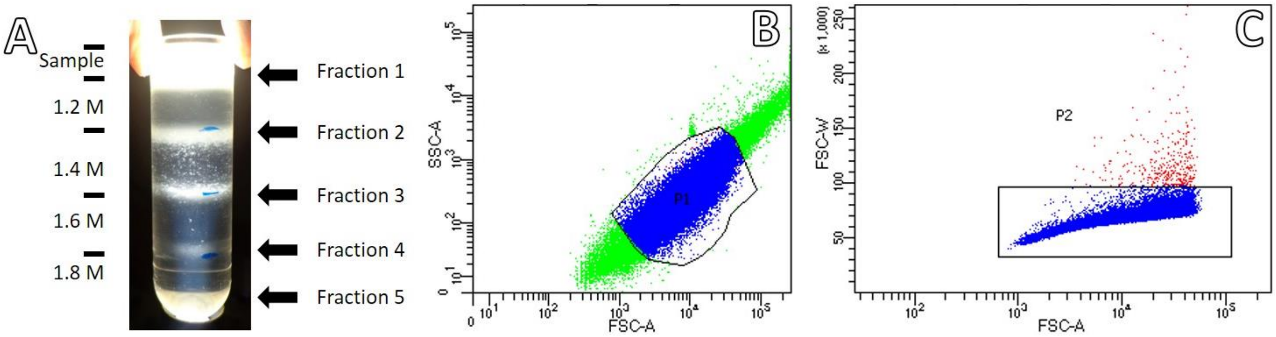

Figure S1. Isolation of amyloid plaque cores (APC). (A) Sucrose gradient (1.0-1.8 M) of APC indicating fractions recovered after ultracentrifugation. Fractions 2 and 3 concentrated by centrifugation. **(B)** Cell sorting, P1 area of selected population. **(C)** Cell sorting, P2 area of recovered population.

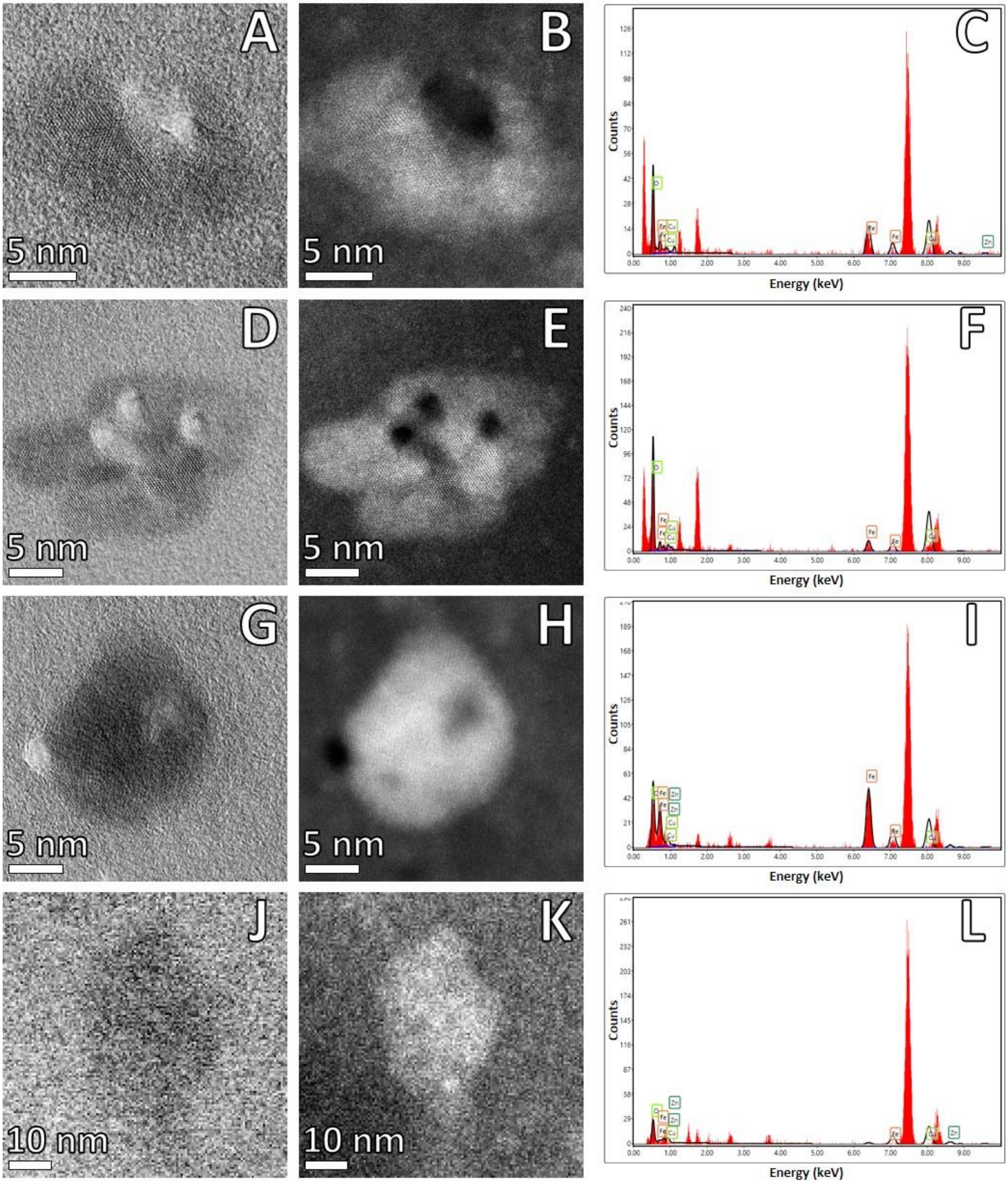

Figure S2. Atomic resolution STEM imaging of particles analyzed by EELS. **(A)** BF-STEM spherical Fe nanoparticle #1. **(B)** HAADF-STEM spherical Fe nanoparticle #1. **(C)** EDX spectra. **(D)** BF-STEM spherical Fe nanoparticle #2. **(E)** HAADF-STEM spherical Fe nanoparticle #2. **(F)** EDX spectra. **(G)** BF-STEM spherical Fe nanoparticle #3. **(H)** HAADF-STEM spherical Fe nanoparticle #3. **(I)** EDX spectra. **(J)** BF-STEM area #4. **(K)** HAADF-STEM area #4. **(L)** EDX spectra. Imaging with JEOL ARM-200F operated in STEM mode at accelerating voltage of 80 kV.

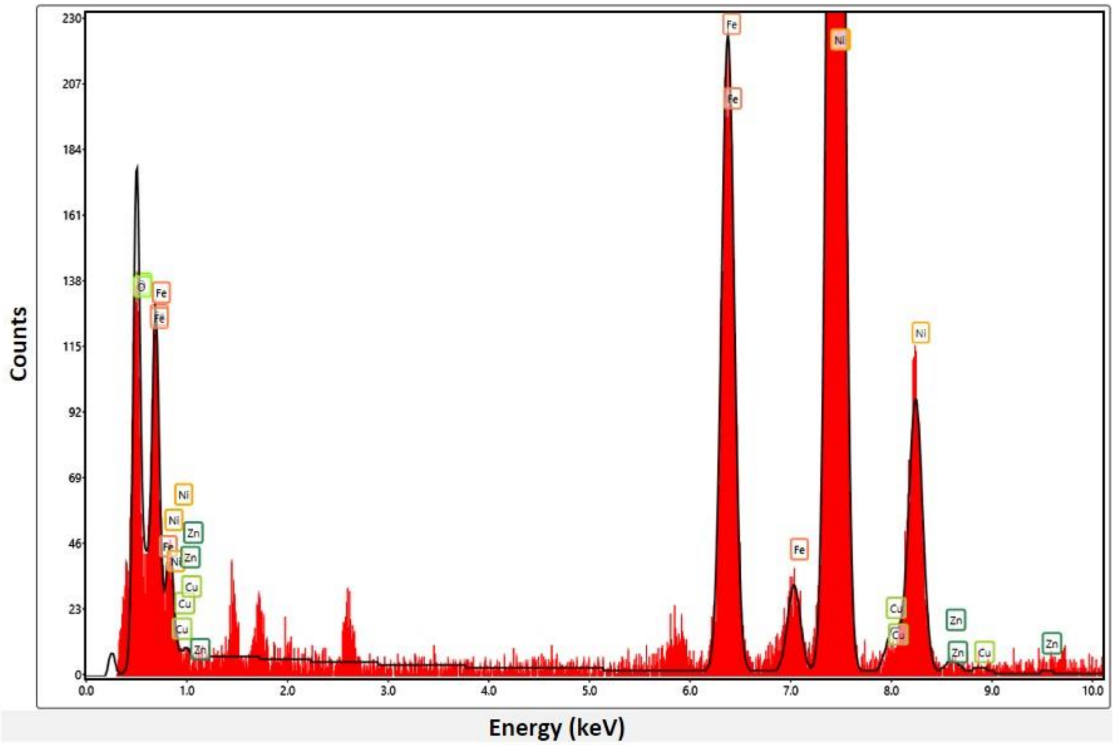

Figure S3. Identification of particles analyzed by EDX microanalysis. EDX spectra indicate location of characteristic X-ray peaks of the elements presented in **Figure 6**: Iron (Fe, red), Oxygen (O, green), Copper (Cu, blue) and Zinc (Zn, yellow). Acquisition with Octane Silicon Drift Detector (SDD) and TEAM EDS Analysis System (EDAX).

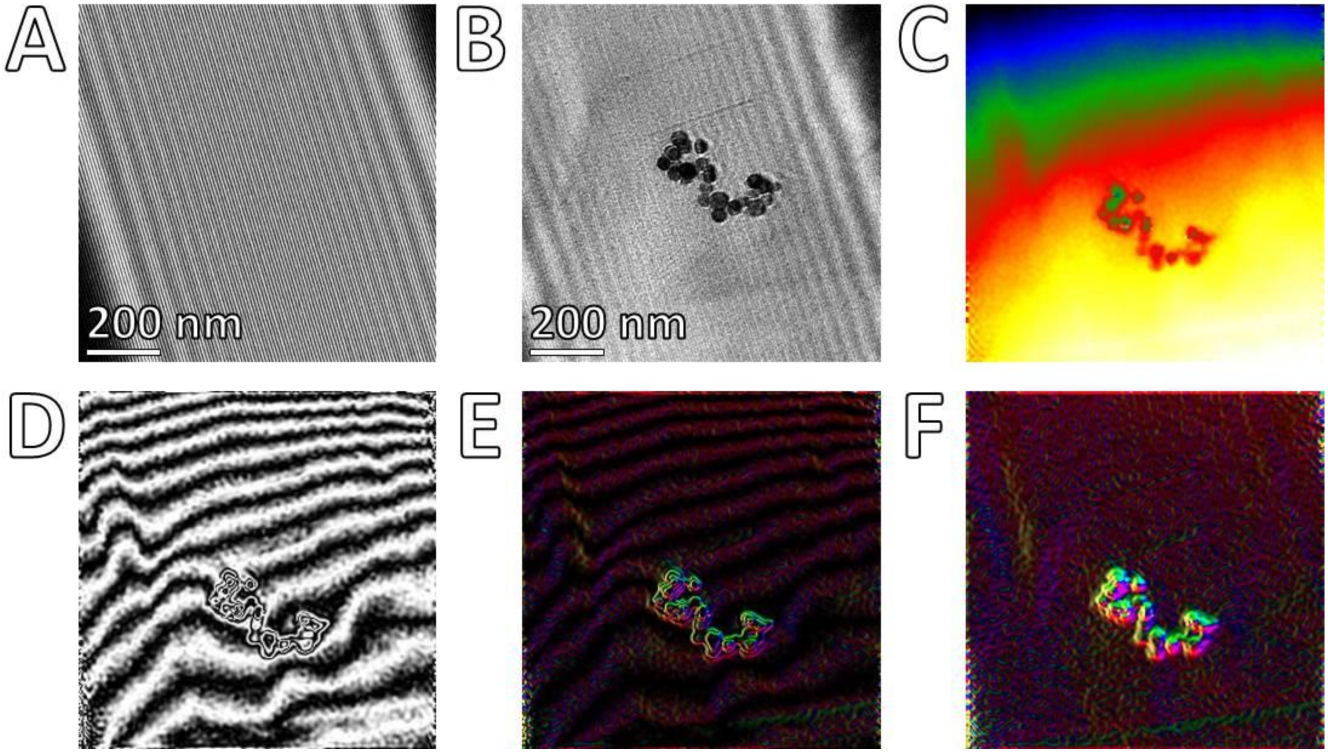

Figure S4. Medium resolution off-axis electron holography. (A) Off-axis electron holography reference diagram, **(B)** Off-axis electron holography object, **(C)** Phase, **(D)** Magnetic contour, **(E)** Magnetic contour, **(F)** Magnetic induction. Imaging with JEOL ARM-200F operated in STEM mode at accelerating voltage of 200 kV.

Micromagnetic calculations for spherical nanoparticles of Fe_3O_4.

Micromagnetic calculations for spherical nanoparticles of Fe_3O_4 at room temperature were carried out based on the dynamic magnetization description given by the Landau-Lifshitz Gilbert equation of motion and its time integration technique by means of finite element method. The magnetic parameters used for simulations were: magnetocrystalline constant K_1=11.0x10^5 J m^{-3}, saturation magnetization M_s=477.7 kA m^{-1} and exchange constant A=0.35 x10^{-11} J m^{-1}. The calculated magnetization corresponding to spherical Fe_3O_4 nanoparticles with diameters D are shown for **(Figure 7-F)** D=70 nm, for which the saturated magnetic state is manifested, with the magnetization pointing along the magnetic anisotropy direction, and **(Figure 7-G)** D=80 nm, for which the transition from single domain particles to multi-domain state is observed, with two distinguishable magnetization directions, one along the magnetic anisotropy direction at the center of the particle, and the other with circular orientation around the middle zone.
